# Characterization of analytical errors in thromboelastography interpretation

**DOI:** 10.1016/j.plabm.2020.e00196

**Published:** 2020-12-15

**Authors:** Tapasyapreeti Mukhopadhyay, Arulselvi Subramanian, Hara Prasad Pati, Renu Saxena

**Affiliations:** a Department of Laboratory Medicine, Jai Prakash Narayan Apex Trauma Centre, All India Institute Medical Sciences, New Delhi, 110029, India; b Room No. 207, Department of Laboratory Medicine, Jai Prakash Narayan Apex Trauma Centre, All India Institute Medical Sciences, New Delhi, 110029, India; c Department of Hematology, All India Institute Medical Sciences, New Delhi, 110029, India; d Indian Society of Hematology & Blood Transfusion, India

**Keywords:** Diagnostic delay, Laboratory error, Patient safety, Turnaround time, TEG misinterpretation

## Abstract

**Introduction:**

Interpretation of Thromboelastography (TEG) curve involves correlating patient’s clinical profile with TEG parameters and the tracing, keeping in mind the potential sources of errors, and hence requires expertise. We aimed to analyse the analytical errors in TEG interpretation due to paucity of literature in this regard.

**Material and methods:**

The retrospective study was conducted in an apex trauma center in North India. Five months of data was reviewed by two laboratory physicians, with differences resolved by consensus. Cases with pre-analytical errors, missing data and TEG runs lasting <10 ​min were excluded. The analytical errors were classified into: preventable, potentially preventable, non-preventable, and non-preventable but care could have been improved.

**Results:**

Out of 440 TEG tracings reviewed, 70 were excluded. An analytical error was present in 60/370 (16.2%) tracings. There were six types analytical errors, of which, tracings of severe hypocoagulable states showing k-time ​= ​0 (33.3%) was the commonest, followed by tracings with spikes at irregular intervals (30%). Of all the analytical errors, 29/60 (48.2%) were preventable and 5/60 (8.3%) were potentially preventable.

**Conclusion:**

Analytical variables that lead to errors in TEG interpretation were identified in about one-sixth of the cases and almost half of them were preventable. Awareness about the common errors amongst clinicians and laboratory physicians is critical to prevent treatment delay and safeguard patient safety.

## 1 Introduction

Thromboelastography (TEG) is a viscoelastic haemostatic assay that provides information about the clot dynamics in real-time. The testing process and the interpretation of the result are quite complex. TEG result is represented graphically and numerically, and the final interpretation requires analysis of both, along with correlation with the patient’s medical and treatment history [1,2]. Certain variables encountered in the analytical phase could influence the test results in the post-analytical phase [3]. These can result in misinterpretation or incorrect validation of the final results, an increase in turnaround time of the point-of–care-test and serious delays in clinical decisions. The paucity of published literature on the analytical errors in TEG interpretation prompted us to perform the study.

The study was aimed to identify the common sources of analytical errors in TEG interpretation in a clinical laboratory. The primary objective was to identify analytical errors in the interpretation of TEG and determine their incidence and the secondary objective was to classify the analytical variables in terms of their preventability.

## 2 Material and Methods

This retrospective observational study was conducted in the Department of Laboratory Medicine of an apex trauma center in North India after obtaining institutional ethical clearance (IEC-628/06.09.20198). Our laboratory provides routine as well as round-the-clock laboratory services. A total of 18 medical technologists working in three shifts (Morning-Evening-Night) are trained to perform TEG. All TEG tracings obtained during the period February to June 2019, from the TEM-A automated Thromboelastometer (FramarBiomedica, Rome, Italy) in our laboratory were included in the study. The TEG tracings were reviewed by two laboratory physicians, with differences resolved by consensus. The TEG tracings were interpreted in concurrence with the clinical setting. Clinical data was accessed from the available electronic medical records of the hospital.

The diagnosis of analytical error was made when an error identified in the initial trace was rectified with the help of a corrective measure, and a subsequent error-free trace was obtained. Cases with obvious pre-analytical errors such as low sample volume, clotted or diluted sample, TEG runs lasting less than 10 ​min, missing data, specimens without a barcode, and internal quality control samples were excluded. For preventability, the analytical errors were further classified into the following categories: Preventable, potentially preventable, non-preventable and non-preventable but care could have been improved. The data was exported to Microsoft Excel for analysis. The collected data being descriptive, has been represented as frequency and percentages.

## 3 Results

**Fig. 1:**
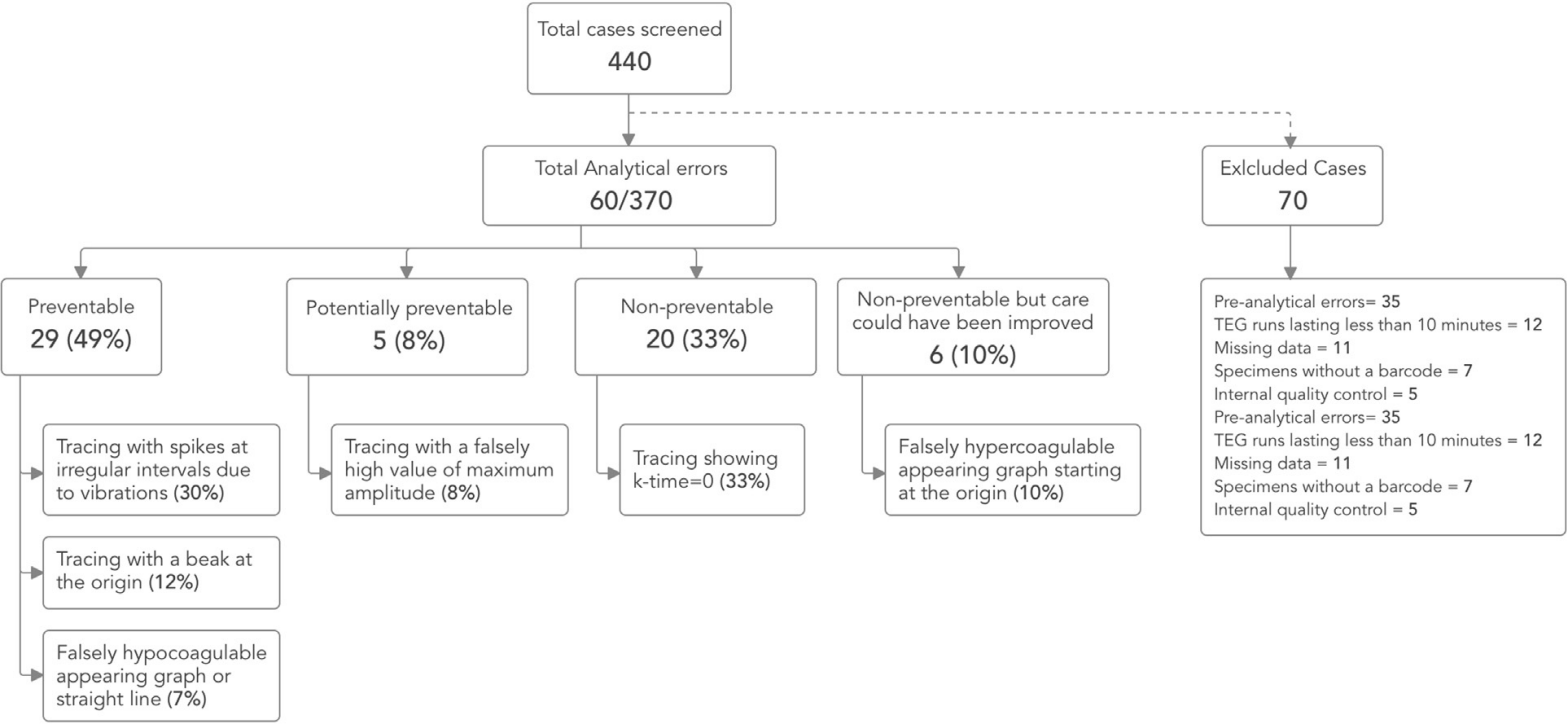
Preventability of analytical errors in TEG tracings.

**Fig. 2:**
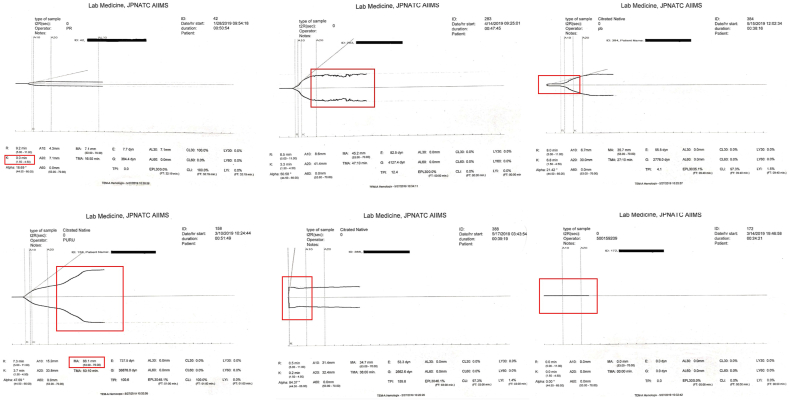
(a–f): (a) False value of k-time observed in severe hypocoagulable state (b) Spikes in TEG tracing due to vibrations in the surrounding (c) Inappropriate placement of the cup in the instrument resulting in a beak-shaped TEG tracing (d) False high value of maximum amplitude due to evaporation of sample during the run (e) Failed auto-calibration before a sample run appears as a hypercoagulable state (f) Delayed clot initiation due to the use of old calcium chloride leading to a hypocoagulable graph or straight line.

A total of 440 TEG studies were performed during the study duration. After screening, 70 studies were excluded based on the exclusion criteria. Out of the remaining 370 TEG tracings, 60 (16.2%) had an analytical error (Fig. 1). The types of erroneous TEG tracings, their respective sources of errors and their nature in terms of preventability (Table 1) are described below:

(i) False suggestion of hypercoagulable state by tracing showing k-time ​= ​0 (Fig. 2a) in patients in very severe hypocoagulable state was the commonest error and was seen in 20/60 (33.3%) cases. This occurred due to the default instrument configuration and was the only non-preventable analytical error.
(ii) Tracing with spikes at irregular intervals (Fig. 2b) due to vibrations near the instrument during the sample run. This was the most frequently occurring preventable error and was observed in 18/60 (30%) cases.
(iii) Tracing with a beak at the origin (Fig. 2c) due to incorrect placement of the reaction cup in the instrument socket. This was the second most common preventable source of analytical error and was observed in 7/60 (11.7%) cases.
(iv) Tracing with a falsely high value of maximum amplitude (Fig. 2d) due to evaporation of the sample during the testing. This was classified as a ‘potentially preventable’ source of analytical error and was seen in 5/60 (8.3%) cases.
(v) Falsely hypercoagulable appearing graph (Fig. 2e) starting at the origin due to failure of auto-calibration before the TEG run. This was classified as ‘non-preventable but care could have been improved’ and was noted in 6/60 (10%) cases.
(vi) Falsely hypocoagulable appearing graph (Fig. 2f) or straight line that improved after using freshly prepared calcium chloride (not older than 24 ​h) was seen in 4/60 (6.7%) cases and was preventable in nature.

**Table 1:** Characterization of analytical errors in TEG interpretation.

TEG tracing	Sources of analytical error	Preventability of the error	Frequency N (%)
Hypocoagulable state	Default instrument setting to k-time ​= ​0 in very severe hypocoagulable state	Non-preventable	20 (33.3)
	Old calcium chloride	Preventable	4 (6.6)
Hypercoagulable state	Instrument error due to failed auto-calibration	Non-preventable but care could have been improved	6 (10)
	Evaporation of the sample from the reaction cup	Potentially preventable	5 (8.3)
Beak shaped graph	Incorrect placement of the reaction cup	Preventable	7 (11.6)
Spikes	Disturbance in the surrounding environment	Preventable	18 (30)

## 4 Discussion

TEG has become the standard of care in managing patients undergoing liver transplant or cardiothoracic surgery, those on antithrombotic therapy and those having obstetric issues [4,5]. It is also widely used to rationalise the use of blood products in both trauma and non-trauma cases [[6], [7], [8]]. However, error-free and precise interpretation of TEG requires supervised training and experience to avoid misinterpretation which can result in unfavourable patient outcomes [9]. In this context, our study identified the incidence of analytical errors related to TEG to be 16.2%, with the majority of the analytical errors being preventable or potentially preventable.

The commonest analytical error observed in the present study was an isolated abnormality of k-time ​= ​0 in a very severe hypocoagulable state. This extreme value of k-time is erroneous and is a result of the failure of the slope of the graph to reach an amplitude of 20 ​mm after the initiation of the clot formation[2]. As is expected, all the TEG numerical parameters were found to be prolonged in these patients with true hypocoagulable state due to delayed clot formation. Therefore, not correlating the other TEG parameters with the tracing may result in a false interpretation of the hypercoagulable state.

The second most common error encountered was spikes in the TEG tracings occurring due to vibration in the proximity of the instrument. Maintaining a vibration-free environment by avoiding any mechanical disturbance to the instrument or the platform on which it is placed can ensure the formation of a smooth curve. Standard procedures that apply to handling and maintenance of the analytical testing equipment in compliance with ISO/IEC 17025:2017 and the Association of Official Analytical Chemists (AOAC) requirements are readily available[10].

The third most common source of error was the wrongly placed reaction cup in the instrument. The incorrect placement of the reaction cup in the specified socket results in a reduced space between the cup and the pin, seen as a tracing with a beak-shaped starting at the origin. This error can be avoided by having trained and competent laboratory personnel dedicated to operating TEG. Commission on Office Laboratory Accreditation (COLA) guidelines mandate competency assessments every six months for the first year of employment and annually thereafter whereas Clinical Laboratory Improvement Amendments of 1988 (CLIA, 1988) recommends an annual assessment of the skills of technical personnel [[11], [12], [13]].

The next commonly encountered error was a falsely hypocoagulable graph due to the quality of calcium chloride used in the testing process [14]. Re-running such a sample with freshly prepared calcium chloride (not older than 24 ​h after its reconstitution) immediately corrects the test result.

Another factor found to cause an analytical error was the evaporation of the plasma. A graph representing a hypercoagulable state with a falsely high value of the maximum amplitude denoting higher clot strength was observed. This can be prevented by optimising and regularly monitoring the ambient room temperature and humidity[15].

Lastly, the failure to auto-calibrate before testing a sample led to the formation of a typical wide opening graph starting from the origin. Before every sample run, the instrument takes a few seconds to auto-calibrate. An inexperienced person may misinterpret the tracing as a hypercoagulable state due to the wide maximum amplitude with or without a shorter k-time and a large alpha angle. A good machine maintenance culture by the operators can ensure a lower frequency of instrument breakdown.

Since multiple variables are involved in the outcome of the TEG test results, it is susceptible to errors. Many a times in busy laboratories, the result is dispatched without the knowledge of the laboratory physician due to clinician’s request for an immediate report. Because of this, the report despatched is fraught with potential danger to patient care. Hence, a better understanding of the working principle of the test and developing expertise to operate TEG may aid in minimising the occurrence of errors, ensure reproducibility of the test results, and help troubleshoot and plan corrective strategies to overcome them.

A few more potential analytical errors that could be possibly identified in real-time, may have been missed as our study has the limitation of being a retrospective analysis. Thus, the frequency observed may also vary in a prospective study and in different centers.

## 5 Conclusion

This paper highlights the common analytical errors that may lead to misinterpretation of TEG results, with most of them being preventable. Clinicians who largely depend upon TEG for the management of patients should be familiar with the common analytical errors. Knowledge amongst laboratory personnel regarding the potential sources of errors is essential to ensure the reproducibility of the test results, maximise patient safety and prevent serious delays in clinical decisions.

## Author statement

Tapasyapreeti Mukhopadhyay: Conceptualization, Design, Definition of intellectual content, Literature search, Clinical studies, Experimental studies, Data acquisition, Formal analysis, Statistical analysis, Manuscript preparation, Writing – review & editing, Manuscript review, Arulselvi Subramanian: Conceptualization, Design, Definition of intellectual content, Literature search, Writing – review & editing, Manuscript review, Guarantor, Hara Prasad Pati: Design, Definition of intellectual content, Writing – review & editing, Manuscript review, Renu Saxena: Design, Definition of intellectual content, Writing – review & editing, Manuscript review

## Conflicting interest

Nil.
